# The Wyckoff positional order and polyhedral intergrowth in the M_3_B_2_- and M_5_B_3_-type boride precipitated in the Ni-based superalloys

**DOI:** 10.1038/srep07367

**Published:** 2014-12-08

**Authors:** X. B. Hu, Y. L. Zhu, N. C. Sheng, X. L. Ma

**Affiliations:** 1 Shenyang National Laboratory for Materials Science, Institute of Metal Research, Chinese Academy of Sciences, Wenhua Road 72, 110016 Shenyang, China; 2 Superalloys Division, Institute of Metal Research, Chinese Academy of Sciences, Wenhua Road 72, 110016 Shenyang, China

## Abstract

Ni-based single superalloys play a crucial role in the hottest parts of jet engines. However, due to the complex geometry and macro-segregation during the solidification process, the cast defect such as stray grains is inevitable. Therefore, the transient liquid phase (TLP) bonding which can join several small single crystalline castings together is gradually believed to be an effective method for improving the yields of production of the complex components. The melting point depressant element B is always added into the interlayer filler material. Consequently, borides including the M_3_B_2_ and M_5_B_3_ phase usually precipitate during the TLP bonding process. So a comprehensive knowledge of the fine structural characteristics of the borides is very critical for an accurate evaluation of the TLP bonding process. In this work, by means of the aberration-corrected transmission electron microscopy, we show, at an atomic scale, the Wyckoff positional order phenomenon of the metal atoms in the unit cell of M_3_B_2_- and M_5_B_3_-type boride. Meanwhile, the defect along the (001) plane of the above two types of boride are determined to be the polyhedral intergrowth with complex configurations.

Single crystal (SX) turbine blades are widely used to increase the efficiency of advanced aero-engines in the past a few decades^1^,^2^. It is universally accepted that the temperature capability of a superalloy increases with the overall contents of solid solution strengthening elements such as W, Mo, Ta, Re and Ru. However, owing to the complex geometry of turbine blades and the relatively lower diffusivity of the heavy elements, macro-segregation is unavoidable during the directionally solidified process. Thus, the cast defects such as stray grains, small-angle grain boundary and freckles frequently occur^3^,^4^. These unexpected defects result in a yield decrease and a cost increase. In order to improve the yields and to decrease the cost, the transient liquid phase (TLP) bonding which joins several smaller SX castings together gradually becomes an effective and promising method^5^,^6^. As a melting depressant element (MPD), boron (B) is usually introduced into the jointing materials. During the TLP bonding process, a great number of borides tend to precipitate in vicinity of the joint because of diffusion of B into the parent material from the molten filler^7^,^8^,^9^,^10^. Therefore, a comprehensive knowledge of the microstructural characteristics of the borides is of great importance for better assessing of the TLP bonding process.

Several boride precipitates in superalloy such as M_3_B_2_^11^,^12^,^13^, M_5_B_3_^14^,^15^,^16^ and M_2_B^17^,^18^,^19^ are often observed, where M denotes metal atoms. Nevertheless, the fine structural characteristics in M_3_B_2_ and M_5_B_3_ phases are known little. Based on X-ray diffraction (XRD) analysis, Beattie^11^ proposed the concept of ordered occupation phenomenon for metal atoms in M_3_B_2_-type boride. However, various precipitates in superalloy usually coexist and the total content of the precipitates is always so little that XRD cannot detect. In contrast, electron diffraction including rotation electron diffraction (RED)^20^,^21^,^22^ is able to determine the crystallographic information from rather small domains. Particularly, the recently developed aberration-corrected scanning transmission electron microscopy (STEM) is capable of displaying the morphological information at a sub-angstrom resolution. This development enables many existing structural problems to be directly imaged at an atomic scale^23^,^24^,^25^,^26^,^27^,^28^,^29^,^30^,^31^,^32^,^33^,^34^,^35^. In this work, we extensively investigate, by means of aberration-corrected high angle annular dark field (HAADF) Z-contrast STEM imaging mode, the structural details in the M_3_B_2_- and M_5_B_3_-type borides that precipitate during the TLP bonding process. We propose the polyhedral stacking of square anti-square prism and trigonal prism to rationalize the observed microstructural characteristics.

## Results

### The structure and composition of the M_3_B_2_-type boride

**Figure 1:**
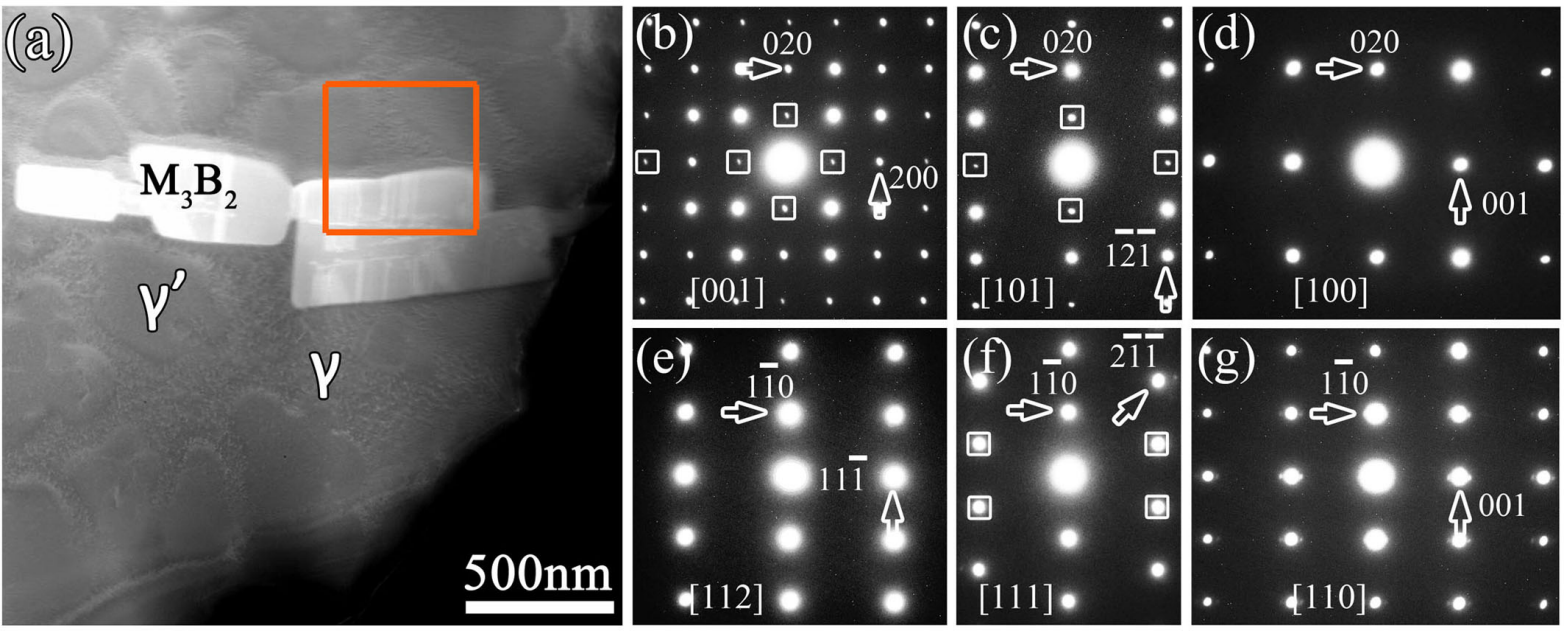
Structural determination of the M_3_B_2_-type boride. (a) HAADF image displaying the M_3_B_2_ phase that precipitated at grain interior during the TLP bonding process. The γ, γ′ and M_3_B_2_ phase are denoted. The orange square frame indicates EDS image-scan area as shown in Fig. 2. (b)–(g) Electron diffraction patterns (EDPs) of M_3_B_2_ phase, they are indexed as [001], [101], [100], [112], [111] and [110] zone-axis, respectively. The M_3_B_2_-type boride with space group of *P4/mbm* is determined according to the EDPs. Because of dynamic diffraction, the forbidden reflections occur and some are marked with square frames.

**Figure 2:**
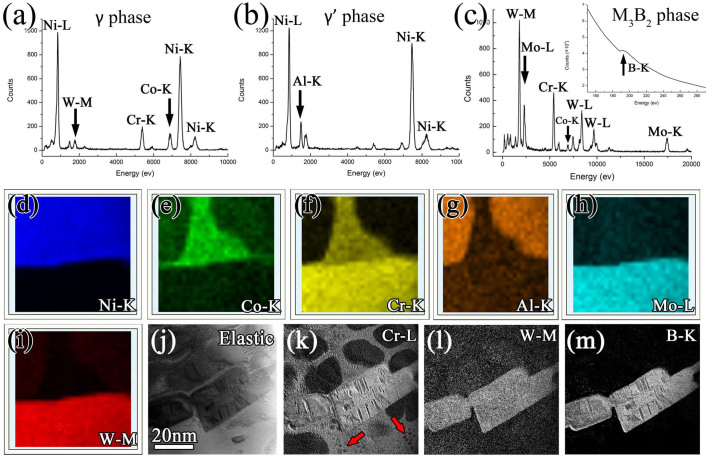
Composition information of the matrix (γ/γ′) and M_3_B_2_-type boride. (a)–(c) STEM-EDS spectrum displaying the chemical composition of γ, γ′, and M_3_B_2_ phase, respectively. The top-right inset in (c) is a TEM-EELS spectrum for absorption edge of B-K at 188eV indicated with an arrow, which was acquired from the M_3_B_2_ phase. (d)–(i) Elemental maps of Ni-K, Co-K, Cr-K, Al-K, Mo-L and W-M, respectively. The EDS image-scan area is indicated with an orange frame in Fig. 1a. (j)–(m) Energy filtered TEM image corresponding to zero loss peak map, Cr-L, W-M, and B-K, respectively. The arrows in (k) indicate the secondary γ′ precipitates in γ matrix.

In contrast to the TEM mode, the STEM mode is very convenient for micro-chemical analysis. Fig. 1a is an HAADF image revealing a lath-shaped M_3_B_2_ precipitation in the matrix during the TLP bonding process. Compared with the matrix (γ/γ′), M_3_B_2_ phase shows brighter contrast under HAADF imaging mode due to the relatively higher average atomic number (Z). The framed area corresponds to the EDS image-scan zone and the composition is shown in Fig. 2d–i. For a detailed structural determination of M_3_B_2_ phase, it is necessary to obtain a series of electron diffraction patterns (EDPs) by large-angle tilting. The acquired EDPs from the M_3_B_2_ phase are shown in Fig. 1b–g, which correspond to [001], [101], [100], [112], [111] and [110] zone-axis, respectively. Due to the tilting angle limitation of the used TEM, the EDPs were acquired from more than one grain. Moreover, at least two EDPs were acquired in each grain. All the *hkl* reflections in [001], [101], [112], [111] and [110] EDP are present, which imply a primitive unit cell. In the [100] EDP, only *0kl* reflections with *k* even appear agreeing with *b*-glide on (100) plane. Based on extinction rules in these EDPs, lattice type of M_3_B_2_ phase is derived as a primitive tetragonal structure with the space group of P4/mbm and lattice parameters of *a* = 0.57 nm and *c* = 0.30 nm. It has a D5_a_-type structure (Strukturbericht notation) based on the information of atomic occupations in M_3_B_2_ phase.

The energy-dispersive spectrum (EDS) shown in Fig. 2a–c displays the compositional information of γ, γ′ and M_3_B_2_ phase, respectively. It is seen that the γ phase is composed of Ni, Co, Cr, W; γ′ of Ni, Al, Cr, W and M_3_B_2_ of W, Cr, Mo, Ni, Co. The boron-K edge peak from the M_3_B_2_ phase is given by the electron energy loss spectrum (EELS) shown as top-right inset in Fig. 2c. Moreover, EDS image-scanning is performed on the framed area in Fig. 1a to display the element distribution in the boride and the matrix. The element maps of Ni, Co, Cr, Al, Mo and W are shown in Fig. 2d–i, respectively. It is seen that, compared with γ phase, γ′ phase is rich of Al and W and lack of Co and Cr. Ni and Mo are homogeneously distributed in γ and γ′. Moreover, compared with the matrix (γ/γ′), M_3_B_2_ precipitate is abundant in W, Cr, and Mo. To detect the light element distribution of boron, energy filtered TEM (EFTEM) was performed. Fig. 2j is a zero-loss peak map image with elastic electrons showing the overall distribution of the matrix and boride. Fig. 2k and l correspond to Cr-L filtered and W-M filtered map, which exhibit the similar contrast difference between the matrix and the boride as that shown in EDS maps (Fig. 2f and i). The nano-particles marked with arrows in Fig. 2k are secondary γ′ phases precipitated during the furnace cooled process. The B-K map in Fig. 2m shows B segregation in the boride. Ti is not detected both in the matrix and in the M_3_B_2_ precipitates due to its low volume in the present alloy.

### The structure and composition of the M_5_B_3_-type boride

**Figure 3:**
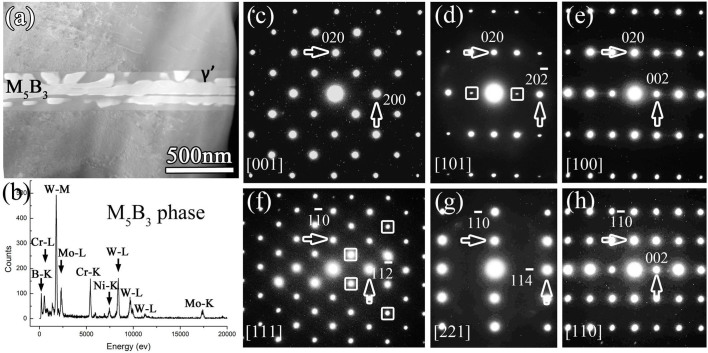
Structural determination and composition information of the M_5_B_3_-type boride. (a) HAADF image showing the M_5_B_3_ phase that precipitated at grain interior during the TLP bonding process. (b) STEM-EDS spectrum showing the chemical composition of M_5_B_3_ phase. (c)–(h) EDPs of M_5_B_3_ phase, indexed as [001], [101], [100], [111], [221], and [110] zone-axis, respectively. The M_5_B_3_-type boride with space group of *I4/mcm* is determined according to the EDPs. Because of dynamic diffraction, the forbidden reflections occur and some are marked.

Fig. 3a is an HAADF image revealing the M_5_B_3_ phase that precipitated in the matrix during the TLP bonding process. The brighter contrast of M_5_B_3_ phase indicates the relatively higher average atomic number (Z) compared with the matrix. The composition of M_5_B_3_ precipitates is shown in Fig. 3b. It is seen that the M_5_B_3_ phase possesses the same constitution as M_3_B_2_ phase. For the sake of convenience, we classify the metal elements into two groups: heavy element with large atomic radius (such as major W, minor Mo) and relatively lighter element with smaller atomic radius (such as major Cr, trace Co, Ni). Although M_3_B_2_ and M_5_B_3_ phase share similar EDS peak shape, they are distinguishable by fine analysis of the EDS data shown in Fig. 2c and Fig. 3b. We take element W as a representative of large atomic radius group and Cr small atomic radius group, respectively. In Fig. 2c, the value of H_W-M_/H_Cr-K_ is approximately 2.1, where H_W-M_ and H_Cr-K_ is the peak height of W-M and Cr-K, respectively. But in Fig. 3b, the value of H_W-M_/H_Cr-K_ is approximately 3.1. Since the peak height of each element is associated with the content, we propose that the M_5_B_3_-type boride is richer of heavy elements with large atomic radius in contrast to the M_3_B_2_-type boride.

To reveal the structure of M_5_B_3_-type precipitate, a series of EDPs are acquired as shown in Fig. 3c–h, which correspond to [001], [101], [100], [111], [221] and [110] zone-axis, respectively. All the *hkl* reflections in Fig. 3c–h EDPs with *h+k+l* = even appear implying a body-centered unit cell. In the [100] EDP, only *0kl* reflections with *l* even appear agreeing with *c*-glide on (100) plane. Based on extinction rules in these EDPs, the lattice type of the M_5_B_3_ phase is derived as a body-centered tetragonal structure with the space group of I4/mcm and lattice parameters of *a* = 0.57 nm and *c* = 1.04 nm. It has a D8_1_-type structure (Strukturbericht notation) based on the information of atomic occupations in M_5_B_3_ phase.

### The crystallographic considerations

To establish the structural relationships of M_3_B_2_ (D5_a_), M_5_B_3_ (D8_1_) and M_2_B (C16 for Strukturbericht notation), detailed crystallographic data of binary borides with the above structures are given in Table 1. It is seen that the parameter of *a* in the above three tetragonal lattices are nearly the same. Moreover, c_M5B3_ nearly equals to 2c_M3B2_ and c_M2B_.

**Figure 4:**
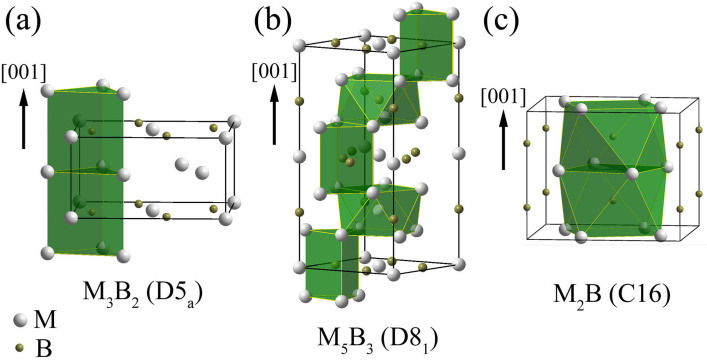
Polyhedron stacking in M_3_B_2_-, M_5_B_3_- and M_2_B-type boride. (a)–(c) Schematic illustrations showing the atomic configurations in M_3_B_2_ (D5_a_), M_5_B_3_ (D8_1_), M_2_B (C16) phase, respectively. The basic polyhedron of trigonal prism and anti-square prism in each structure are shadowed. The [001] stacking direction in each structure is arrowed. For single polyhedron, the metal atoms M occupy the vertex positions and the B atom occupies the central position.

It is known that the structure of boride can be described by the stacking of polyhedron with metal atoms in vertexes and B atoms in center for simplification^19^,^36^,^37^,^38^,^39^. Therefore, in order to schematically illustrate the polyhedron stacking, pictorial diagrams of M_3_B_2_, M_5_B_3_ and M_2_B phase are shown in Fig. 4a–c, respectively. The unit cells are indicated by the black frames and the polyhedrons are shadowed. By introducing the concept of polyhedron stacking in borides, structural analysis can be greatly simplified. It is clearly seen that M_3_B_2_-type boride is composed of trigonal prism layers stacking along [001]_M3B2_ direction as shown in Fig. 4a, while M_2_B-type boride is composed of anti-square prism layers stacking along [001]_M2B_ direction as shown in Fig. 4c. In the case of M_5_B_3_-type boride, it is composed by alternatively arranged trigonal prism layers and anti-square prism layers stacking along [001]_M5B3_ direction. We here introduce a simplified description for basic polyhedral units: T and T′ indicate trigonal prisms and A and A′ indicate anti-square prisms. Then, the polyhedron stacking along [001] direction for M_3_B_2_, M_2_B and M_5_B_3_ can be simplified as TTT or T′T′T′, AA′AA′, and AT′A′TAT′, respectively. The T′ and A′ indicate the polyhedrons which have a rotation approximately 36.7° compared with T and A along the [001] stacking direction. The relationship of the lattice parameter for the above three structures actually results from the close relationship among constituent polyhedron stacking. In addition, the close relationship between these three borides is expected to induce an intergrowth of these phases with the crystallographic orientation relationship of [100]_M3B2_//[100]_M2B_//[100]_M5B3_, [010]_M3B2_//[010]_M2B_//[010]_M5B3_ and [001]_M3B2_//[001]_M2B_//[001]_M5B3_.

### The ordered occupation of metal atoms in M_3_B_2_-type boride

**Figure 5:**
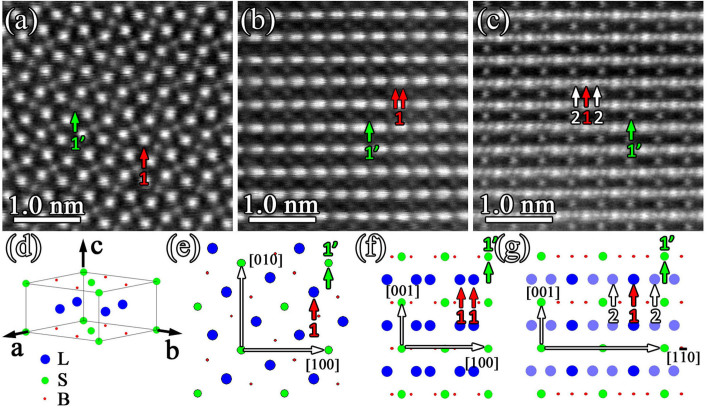
Ordered occupation of metal atoms in M_3_B_2_-type boride. (a)–(c) Atomic HAADF image taken along [001]_M3B2_, [100]_M3B2_ and [110]_M3B2_ direction, respectively, reveals the ordered occupation of metal atoms M in M_3_B_2_ phase. (d) Atomic configuration in the unit cell of M_3_B_2_ phase with ordered occupation of metal atoms. The blue balls represent large metal atoms (designated as L) such as W and Mo. The green balls represent relatively smaller metal atoms (designated as S) such as Cr, Co and Ni. The B atoms are indicated by red balls. The ordered M_3_B_2_ phase should be treated as a ternary boride with the chemical formula of L_2_SB_2_. (e)–(g) Structural projection of ordered M_3_B_2_ phase (L_2_SB_2_) along [001]_M3B2_, [100]_M3B2_ and [110]_M3B2_ direction, respectively. The main directions in each zone-axis are indicated with arrows. The atomic column marked by 1 and 1′ in (a)–(c) and (e)–(g) have the same atomic number density (AND) along the projected direction but with different atomic occupations. The upward arrows marked at column 1 and 2 in (c) and (g) indicate the same atomic occupation but with different ANDs along the projected direction.

As seen in above sections, M_3_B_2_-type boride possesses the D5_a_ structure and contains more than one metal element. Meanwhile, the metal atoms can be classified into two groups, namely, heavy elements with large atomic radius such as major W, minor Mo and relatively lighter elements with smaller atomic radius such as major Cr, trace Co, Ni. In order to obtain the atomic occupation of metal atoms in M_3_B_2_ phase, atomic scale Z-contrast images are acquired. Fig. 5a is an atomic HAADF image projected along the four-fold [001]_M3B2_ direction of the M_3_B_2_ phase. Here, the contrast difference of the atomic columns can be divided into two groups, as indicated by the red (marked as 1) and green (marked as 1′) arrows. Considering the same atomic number density (AND) of atomic column 1 and 1′ along [001]_M3B2_ direction for D5_a_ structure, contrast should be the same if metal atoms distribute randomly in the unit cell. However, it is not the case here. The contrast difference between atomic column 1 and 1′ in Fig. 5a indicates that metal atoms distribute in an ordered manner. Based on above information and the atomic configuration in D5_a_ structure, the unit cell of M_3_B_2_-type boride with ordered metal atoms is given in Fig. 5d where blue balls represent large metal atoms (designated as L) such as W, Mo and green balls represent small metal atoms (designated as S) such as Cr, Co, Ni. Then the M_3_B_2_ phase should be treated as a ternary boride with the chemical formula of L_2_SB_2_. Fig. 5e displays a structural projection along [001]_M3B2_ direction of the ordered M_3_B_2_ phase (L_2_SB_2_). To further confirm the ordering in M_3_B_2_ phase, atomic HAADF images along the other two major directions of tetragonal lattice, namely [100]_M3B2_ and [110]_M3B2_ direction, are shown in Fig. 5b and Fig. 5c, respectively. In Fig. 5b, two different contrasts of the atomic columns can be identified as indicated by red (marked as 1) and green (marked as 1′) arrows. Although the AND of atomic column 1 and 1′ in Fig. 5b are the same along [100]_M3B2_ direction, the atomic column 1 is occupied by large metal atoms and atomic column 1′ by small metal atoms. Thereby, the atomic column 1 has brighter contrast than atomic column 1′. The structural projection along [100]_M3B2_ direction of the ordered M_3_B_2_ phase is shown in Fig. 5f. In Fig. 5c, three different contrasts for the atomic columns can be seen as highlighted with red (marked as 1), green (marked as 1′) and white (marked as 2) arrows. The atomic column 1 and 1′ in Fig. 5c have the same AND along [110]_M3B2_ direction. But the atomic column 1 is occupied by large metal atoms and the atomic column 1′ by small metal atoms. Thus the atomic column 1 has brighter contrast than the atomic column 1′. However, for the atomic column 1 and 2 in Fig. 5c, although they are all occupied by large metal atoms, the AND of the atomic column 1 is twice as that of the atomic column 2. So, the atomic column 1 has brighter contrast than the atomic column 2 in Fig. 5c. The structural projection along [110]_M3B2_ direction of the ordered M_3_B_2_ phase is shown in Fig. 5g, where the shadowed atomic column 2 indicates half AND of the atomic column 1. Such an ordering of metal atom occupation in M_3_B_2_ phase was proposed in an XRD study^11^.

### The ordered occupation of metal atoms in M_5_B_3_-type boride

**Figure 6:**
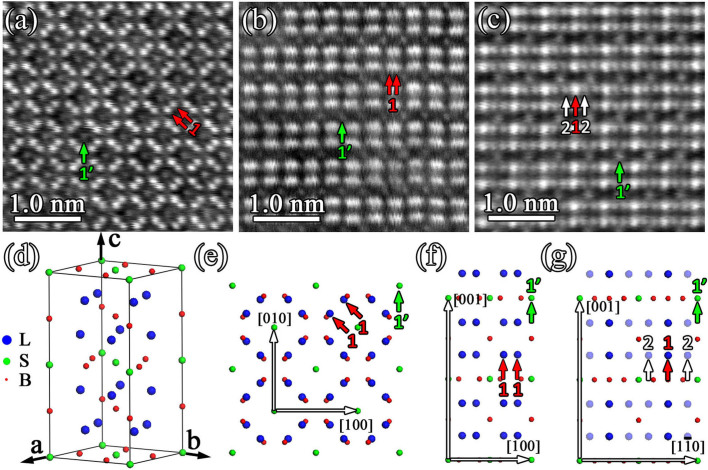
Ordered occupation of metal atoms in M_5_B_3_-type boride. (a)–(c) Atomic HAADF image taken along [001]_M5B3_, [100]_M5B3_ and [110]_M5B3_ direction, respectively, reveals the ordered occupation of metal atoms M in M_5_B_3_ phase. (d) Atomic configuration in the unit cell of M_5_B_3_ phase with ordered occupation of metal atoms. The blue balls represent large metal atoms (designated as L) such as W and Mo. The green balls represent small metal atoms (designated as S) such as Cr, Co and Ni. The B atoms are indicated by red balls. The ordered M_5_B_3_ phase should be treated as a ternary boride with the chemical formula of L_4_SB_3_. (e)–(g) Structural projection of ordered M_5_B_3_ phase (L_4_SB_3_) along [001]_M5B3_, [100]_M5B3_ and [110]_M5B3_ direction, respectively. The main directions in each zone-axis are indicated with arrows. The atomic column marked by 1 and 1′ in (a)–(c) and (e)–(g) have the same atomic number density (AND) along the projected direction but with different atomic occupations. The upward arrows marked at column 1 and 2 in (c) and (g) indicate the same atomic occupation but with different ANDs along the projected direction.

Similarly, to display the atomic occupation of metal atoms in M_5_B_3_-type boride, atomic scale Z-contrast imaging is performed. Fig. 6a is an atomic HAADF image obtained along the four-fold [001]_M5B3_ direction of the M_5_B_3_ phase. In Fig. 6a, contrast difference of the atomic columns can be classified into two groups, as indicated by red (marked as 1) and green (marked as 1′) arrows. Considering the same AND of the atomic column 1 and 1′ along the [001]_M5B3_ direction in D8_1_ structure, the contrast difference between the atomic column 1 and 1′ in Fig. 6a should be ascribed to the ordered distribution of metal atoms in M_5_B_3_-type boride. The unit cell of M_5_B_3_ with ordered metal atoms is given in Fig. 6d where blue balls represent large metal atoms (designated as L) and green balls represent small metal atoms (designated as S). Therefore, the ordered M_5_B_3_ phase should be treated as a ternary boride with the chemical formula labeled as L_4_SB_3_. Fig. 6e displays the structural projection of the ordered M_5_B_3_ phase (L_4_SB_3_) along [001]_M5B3_ direction. For more details, atomic HAADF images along the other two major directions of the tetragonal lattice, namely, [100]_M5B3_ and [110]_M5B3_ direction, are shown in Fig. 6b and c, respectively. In Fig. 6b, two different contrasts of the atomic columns can be seen as indicated by the red (marked as 1) and green (marked as 1′) arrows. In spite of the same AND of the atomic column 1 and 1′ in Fig. 6b, the atomic column 1 is occupied by large metal atoms and atomic column 1′ by small metal atoms. Thus, the atomic column 1 has brighter contrast than the atomic column 1′. The structural projection along [100]_M5B3_ direction of the ordered M_5_B_3_ phase is shown in Fig. 6f. In Fig. 6c, the contrast difference is proposed to result from different atomic occupations taking into account of the same AND of the atomic column 1 and 1′ along [110]_M5B3_ direction. That is to say, the atomic column 1 is occupied by large metal atoms and the atomic column 1′ is occupied by small metal atoms. But the contrast difference where the same large metal atoms occupy the atomic column 1 and 2 is proposed to result from the different ANDs. The AND of the atomic column 1 is twice as that of the atomic column 2 along [110]_M5B3_ direction. The structural projection along [110]_M3B2_ direction of the ordered M_5_B_3_ phase is shown in Fig. 6g, where the shadowed atomic column 2 indicates the half AND of atomic column 1.

### Polyhedral intergrowth in the M_3_B_2_- and M_5_B_3_-type boride

**Figure 7:**
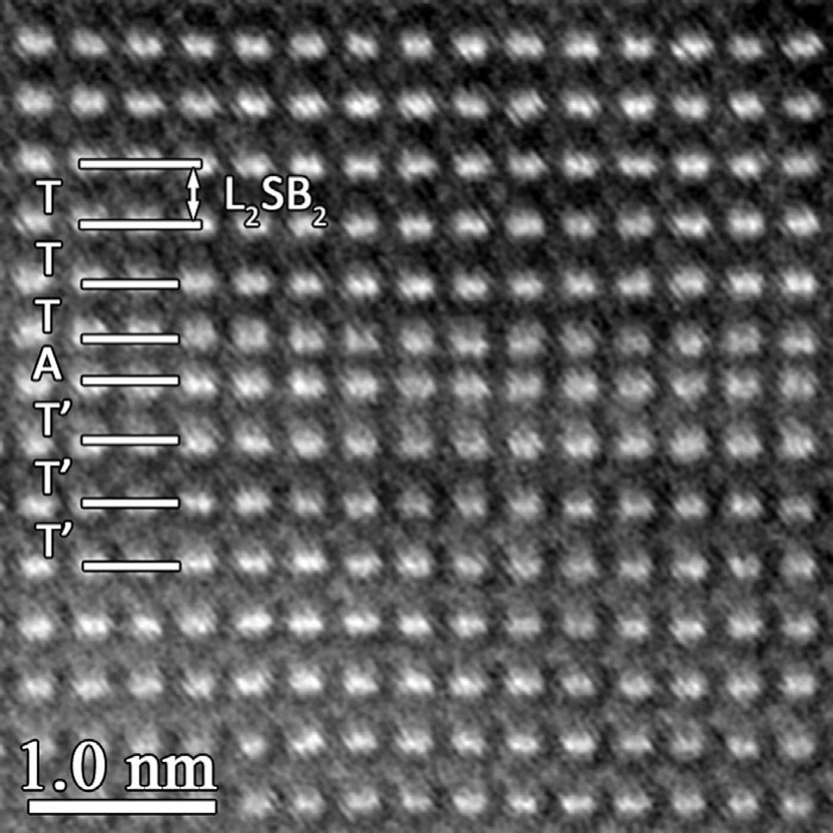
Polyhedral intergrowth in M_3_B_2_-type boride. Atomic HAADF image along [100]_M3B2_ direction showing the polyhedral intergrowth. The stacking sequence of the basic polyhedral units (T, T′, A) along [001]_M3B2_ stacking direction is indicated. Compared with trigonal prism layers designated as T, the trigonal prism layers denoted by T′ have a 36.7° rotation about [001]_M3B2_ direction.

Based on the above crystallographic considerations, it is seen that M_3_B_2_-, M_2_B- and M_5_B_3_-type boride are closely related. Along [001] direction, the M_3_B_2_ and M_2_B phase are completely composed of trigonal prism layers and anti-square prism layers, respectively. Furthermore, considering the fact that the M_5_B_3_ phase is composed of an alternating array of both trigonal prism layer and anti-square prism layer, it is proposed that trigonal prism layer and anti-square prism layer can inter-grow along [001] stacking direction without destroying local coordination environment of the B atoms. Thus, the intergrowth of one or more polyhedral layer along [001] direction inside the M_3_B_2_/M_5_B_3_ phase is expectable. Moreover, due to conservation of polyhedrons consisting of metal and B atoms, the resultant interface by the polyhedral intergrowth is proposed to possess a lower energy. Fig. 7 displays an atomic HAADF image of the M_3_B_2_ phase projected along [100]_M3B2_ direction. The polyhedron stacking along [001]_M3B2_ direction is denoted by the basic polyhedral units (T, T′, A). It is seen that one anti-square prism layer inter-grows within the trigonal prism layers. This defective structure can be described as TTTAT′T′T′ as indicated in Fig. 7. Since an anti-square prism is composed of two sections which have a 36.7 rotation apart from each other along the four-fold direction, the intersection of one anti-square prism (A) leads to the stacking of trigonal prism layer from T to T′.

**Figure 8:**
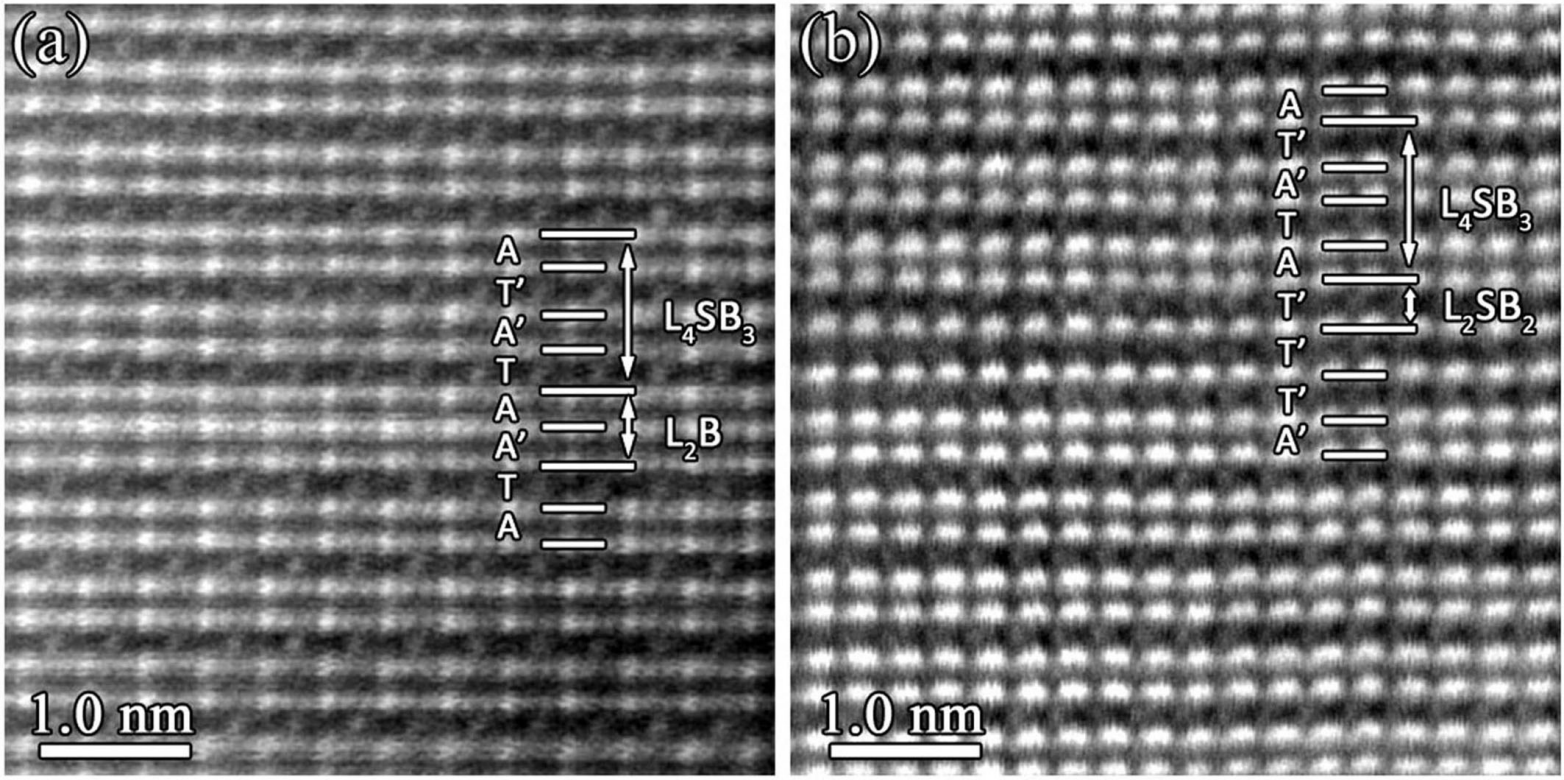
Polyhedral intergrowth in M_5_B_3_-type boride. (a) Atomic HAADF image along [110]_M5B3_ showing the intergrowth of one anti-square prism layer inside the M_5_B_3_ phase. (b) Atomic HAADF image along [100]_M5B3_ displaying the intergrowth of two trigonal prism layers inside the M_5_B_3_ phase. The perfect M_5_B_3_ phase is composed of an alternating array of trigonal prism layers and anti-square prism layers stacking along [001]_M5B3_ direction. The stacking sequence of the basic polyhedral units (T, T′, A, A′) along [001]_M3B2_ stacking direction is indicated. Compared with anti-square prism layers designated as A, the anti-square prism layers denoted by A′ have a 36.7° rotation about [001]_M5B3_ direction.

Fig. 8a is an atomic HAADF image acquired along [110]_M5B3_ direction of the M_5_B_3_-type boride. The polyhedron stacking along [001]_M5B3_ direction is denoted by the basic polyhedral units (T, T′, A, A′), where an intergrowth of an anti-square prism layer is identified. For the sake of simplification, such a defective structure can be described as AT′A′TAA′TA. Due to the local intersection of one anti-square prism layer, a unit cell of M_2_B (L_2_B)-type boride is derived with stacking of AA′. Fig. 8b is an HAADF image of M_5_B_3_ phase projected along [100]_M5B3_ direction. Based on the designation of the basic polyhedral units (T, T′, A, A′), an intergrowth of two trigonal prism layers was identified. This defective structure can be described as AT′A′TAT′T′T′A′ as indicated in Fig. 8b. Due to the local intergrowth of two trigonal prism layers, a small-scale M_3_B_2_ (L_2_SB_3_)-type boride is derived with stacking form of T′T′T′. This polyhedral intergrowth in M_3_B_2_/M_5_B_3_ phase is similar to the intergrowth of various phases in Ti-B system reported by De Graef^38^ and Kooi^39^, respectively.

**Figure 9:**
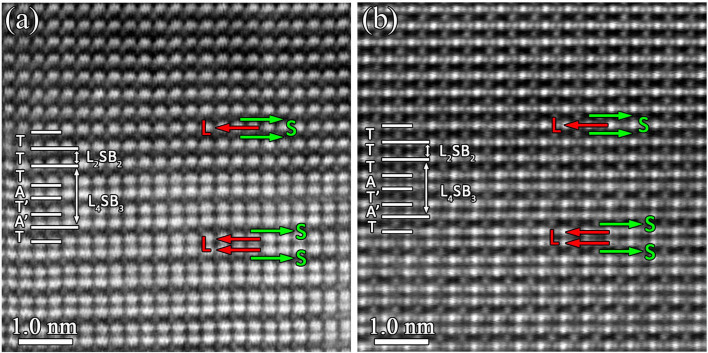
Large-scale intergrowth of the M_3_B_2_- and M_5_B_3_-type boride. Atomic HAADF images along (a) [100]_M3B2_ or [100]_M5B3_ and (b) [110]_M3B2_ or [110]_M5B3_, respectively, reveal the large-scale intergrowth of the M_3_B_2_ and M_5_B_3_ phase. The stacking sequence of basic polyhedral units (T, T′, A, A′) for M_3_B_2_ and M_5_B_3_ along [001] stacking direction is indicated. The red and green arrows in (a)–(b) represent layers of large metal atoms (indicated by L) and layers of small metal atoms (indicated by S), respectively.

Besides the local polyhedral intergrowth, large scale intergrowth of M_3_B_2_ with M_5_B_3_ also occurs in our sample as shown in Fig. 9a–b, which are obtained along the [100]_M5B3_ and [110]_M5B3_ direction, respectively. The interface between M_3_B_2_ and M_5_B_3_ is coherent and lattice misfit dislocation is not identified.

## Discussion

On account of above crystallographic considerations, it is seen that there are two kinds of Wyckoff positions (designated as 16l and 4c) for metal atoms in the D8_1_ structure. They are occupied in order by the metal atoms with different atomic radius. The Wyckoff position 16l is occupied by large metal atoms while Wyckoff position 4c by relatively smaller metal atoms. Since the ordering happens at different Wyckoff positions inside the unit cell, the space group of the ordered structure remains unchanged. This Wyckoff positional order phenomenon is also applicable to the ordering in the M_3_B_2_ phase. The Wyckoff position 4 h in M_3_B_2_ phase is occupied by metal atoms with large atomic radius, while Wyckoff position 2a by metal atoms with relatively smaller atomic radius. The LS_4_B_3_ (LS_2_B_2_) phase with the D8_1_ (D5_a_) structure, in which Wyckoff position 4c (2a) is occupied by large metal atoms and Wyckoff position 16l (4 h) by relatively smaller metal atoms, is not observed in our samples. This selective ordering agrees well with the proposal given by Steeds^40^ that the ordered LS_2_B_2_ phase is not usually seen. In fact, the ordered occupation of the metal atoms at different Wyckoff positions in M_3_B_2_- and M_5_B_3_-type boride may indeed lead to the difference, in contrast to the disordered structure, in the intensity of some diffraction reflections. However, these tiny intensity differences are always screened because the intensity for diffraction spots is usually inaccurate, which is strongly affected by numerous factors such as the sample thickness, exposure time, and deviation for the exact zone-axis orientation.

Although B atoms are invisible in the HAADF images due to its weak scattering ability, the structural characteristics of M_3_B_2_ and M_5_B_3_ phase can be clearly displayed as shown in Fig. 9a–b. Along [001]_M3B2_ direction, there is only one layer of large metal atoms between two layers of small metal atoms. However, in the M_5_B_3_-type boride, two layers of large metal atoms are present between two layers of small metal atoms. This indicates that the fraction of the large metal atoms (or heavy elements) is larger in M_5_B_3_-type boride than that in M_3_B_2_-type boride.

In summary, we have carried out a systemic analysis on the composition and microstructures in the M_3_B_2_- and M_5_B_3_-type boride that precipitated in Ni-based single superalloy during the TLP bonding process. We find that the metal atoms in the lattice of M_3_B_2_ and M_5_B_3_ phase are occupied in an ordered manner. The M_3_B_2_ and M_5_B_3_ phase with ordered occupation can be treated as the ternary boride with the chemical formula of L_2_SB_2_ and L_4_SB_3_, respectively, where L represents metal atoms with large atomic radius and S indicates metals with small atomic radius. This ordering phenomenon is actually Wyckoff positional order inside the unit cell of the M_3_B_2_ and M_5_B_3_ phase. By introducing the concept of polyhedron stacking, the planar defects along (001) of L_2_SB_2_ and L_4_SB_3_ phase are interpreted in terms of the polyhedral intergrowth. Since the interface between the trigonal prism layer and the anti-square prism layer is coherent, the interfacial energy of L_2_SB_2_/L_4_SB_3_ is proposed to be very low, which rationalize the present observation of large-scale intergrowth of L_2_SB_2_ and L_4_SB_3_.

## Methods

### Bulk sample preparation

The 16 mm diameter cylindrical samples with nominal composition of 6.0 Cr, 7.5 Co, 1.2 Mo, 5.8 W, 5.9 Al, 1.1 Ti and balance Ni, in wt% were directionally solidified along [001] direction. Then the 8 mm high and 300 μm wide gap parallel to the [001] direction was cut in the center of the 10 mm high specimen. After cleaned, the specimen with a gap was filled using the gas atomized metal powder with nominal composition of 15.0 Cr, 3.5 B and balance Ni, in wt%. Finally the specimens were bonded in a vacuum furnace at 1200°C under the pressure of 5 × 10^−3^ Pa for 4 h, following by furnace cooled to room temperature. TEM specimens were prepared by cutting, grinding, punching and dimpling to 10 μm. The ion-milling was carried out in a Gatan precision ion polishing system (PIPS) with a liquid-nitrogen-cooled stage for avoiding preferential thinning effects. The specimen was plasma cleaned in the Advanced Plasma System Gatan Solarus 950 before loading in the TEM for preventing the surface contamination.

### Composition analysis and STEM imaging

Micrometer scale structural investigations were performed in the Tecnai G^2^ F30 transmission electron microscope, equipped with a high-angle annular dark-field (HAADF) detector, X-ray energy-dispersive spectrometer (EDS) system and Gatan imaging filter (GIF) system, operated at 300 kV. Electron energy loss spectra (EELS) were collected with 0.3 eV per channel dispersion. The energy filtered TEM (EFTEM) images were recorded by the three-window method. The atomic Z-contrast images were recorded using the aberration corrected scanning transmission electron microscopes Titan^3^™ G^2^ 60-300 fitted with a high-brightness field-emission gun (X-FEG) and double Cs correctors from CEOS, and a monochromator operated at 300 kV. In the scanning transmission electron microscopy (STEM) mode, the convergence angle of the electron beam is approximate 25 mrad, which yields a probe size less than 0.10 nm. And the collection angle is from 50 mrad to 250 mrad. The final resolution approximates 0.08 nm under the STEM mode. According to thickness map acquired from EFTEM, the final thickness used for atomic Z-contrast image ranges from 20 nm to 80 nm. The average background subtraction filter (ABSF) and Wiener filters were used to subtract the signal in the atomic HAADF images arising from the amorphous layer at the surface of the specimen^41^. The Fast Fourier Transform (FFT) pattern of the HR-STEM image was used to determine the exact direction of the projection.

## Author Contributions

The project of fine structural analyses for borides precipitated in superalloy was conceived by X.L.M. and Y.L.Z.; bulk sample was prepared by N.C.S.; thin foils preparation and TEM/STEM observations were performed by X.B.H.; all the authors participated in discussion, interpretation of the data and producing the final version of this paper.

## Figures and Tables

**Table 1:** The structural type, space group, lattice parameters, Wyckoff positions and fractional atom coordinates in W_2_B, V_3_B_2_ and Cr_5_B_3_ phase

Structure	Space group	Unit cell (nm)	Atom	Fractional coordinates		
**W_2_B**	I4/mcm (no. 140)	a = 0.557	Cr (8h)	0.169	0.669	0.000
**C16**		c = 0.474	B (4a)	0.000	0.000	0.250
**V_3_B_2_**	P4/mbm (no. 127)	a = 0.576	V (4h)	0.173	0.673	0.500
**D5_a_**		c = 0.304	V (2a)	0.000	0.000	0.000
			B (4g)	0.388	0.888	0.000
**Cr_5_B_3_**	I4/mcm (no. 140)	a = 0.546	Cr (16l)	0.166	0.666	0.150
			Cr(4c)	0.000	0.000	0.000
**D8_1_**		c = 1.064	B (8h)	0.625	0.125	0.000
			B (4a)	0.000	0.000	0.250
